# Ipriflavone ameliorates intervertebral disc degeneration by inhibiting osteoporosis of vertebral body and pyroptosis of the nucleus pulposus in instability of lumbar spine and diabetic mice

**DOI:** 10.3389/fbioe.2025.1639117

**Published:** 2025-08-05

**Authors:** Qi Sun, Hai-Jing Zhang, Hui Wang, Gang Ji, Ya-Heng Zhao, Gao-Cen Li, Shao-Shi Guo, Lu-Feng Lin, Yu-Jie Jin, Xue-Li Zhang, Xin-Yu Nan, Chang-Cheng Liu, Guo-Bin Liu

**Affiliations:** ^1^ Department of Orthopedic Surgery, The First Hospital of Hebei Medical University, Hebei, China; ^2^ Nursing School, Hebei Medical University, Shijiazhuang, China

**Keywords:** diabetes, pyroptosis, osteoporosis, intervertebral disc degeneration, Ipriflavone

## Abstract

**Background:**

Diabetes mellitus (DM) is a chronic metabolic disease, which can not only induce osteoporosis but also accelerate intervertebral disc degeneration (IVDD). Ipriflavone (IP), as a derivative of isoflavones, can not only control the level of blood glucose, but also improve the regulation of osteoporosis and cartilage extracellular matrix metabolism. However, there is no study on whether IP can effectively improve DM with IVDD.

**Methods:**

Sixty healthy female C57BL/6J mice were randomly assigned into five groups (Sham, Instability of lumbar spine (ILS), streptozotocin (STZ), ILS + STZ and ILS + STZ + IP groups; 12 per group). The body weight, fasting glucose and blood insulin levels were evaluated in each group of mice. The pathology of DM with IVDD was assessed by Micro-CT (μCT), Van Gieson (VG) staining, Alcian blue (Ab) staining, immunohistochemistry, Western blot and real-time polymerase chain reaction (RT-PCR).

**Results:**

IP significantly lowed fasting blood glucose and blood insulin levels. Histomorphological analysis revealed that IVDD was significantly exacerbated by the coexistence of ILS and DM, and markedly alleviated by IP. In details, IP markedly improved osteoporosis and microarchitecture of EP and vertebrae. Furthermore, IP ameliorated the cartilage extracellular matrix degradation, significantly increased Aggrecan and Col II expression and decreased the expression of MMP13 and ADAMTS-4. Moreover, IP inhibited EP sclerosis and NP pyroptosis by decreasing Runx2, Osterix, NLRP3, ASC, N-GSDMD and caspase1 expression.

**Conclusion:**

The coexistence of ILS and DM further aggravates abnormal metabolic pathology and IVDD, which could be retarded by IP, suggesting that IP may be a potential therapeutic target for amelioration of the progression of DM with IVDD.

## Introduction

Low back pain (LBP) is a serious global public health problem affecting 80% of individuals during their lifetime and causing the disability worldwide (Liu et al., 2023). LBP substantially disrupts people’s lives and is a serious economic burden worldwide. Intervertebral disc degeneration (IVDD) is considered the main cause of LBP and other spine-related diseases (Wang et al., 2018). Several factors can contribute to IVDD, including variations in hormone levels, obesity, age, mechanical overloading, nutritional deficiency, oxidative stress, genetics and so on (Zhang Q. et al., 2020; Shi et al., 2023). In addition, Zheng et al. (2020) confirmed that diabetes mellitus (DM) is also an independent risk factor for disc degeneration. It’s worth noting that the rapid development of the global economy and the substantial improvement of people’s living standards has resulted in an increased aging population, and the prevalence of IVDD with DM is increasing annually a problem that cannot be ignored (Mahmoud et al., 2020). Unfortunately, the underlying mechanism of IVDD with DM is poorly understood.

As we all know, the intervertebral disc (IVD) comprises of the central nucleus pulposus (NP), the ligamentous annulus fibrosus (AF), and two (caudal and cranial) cartilaginous endplates (EP) (Ito et al., 2021). The loss of water and proteoglycans from the NP, AF tears, and calcification of the EPs contribute substantially to disc degeneration (Feng et al., 2018). These changes increase the expression of catabolic and inflammatory mediators, including matrix metalloproteinases (MMPs), Aggrecan (Agg), interleukin-1 beta, and tumor necrosis factor-alpha, which can accelerate the degradation of the surrounding extracellular matrix (ECM). Pyroptosis is a novel lytic programmed cell death mechanism triggered by the activation of caspases and inflammasomes. Pyroptosis differs from apoptosis in that it is characterized by the rupture of the plasma membrane and the release of inflammatory mediators, accelerating the destruction of the ECM (Galluzzi et al., 2018). With the increasing interest in pyroptosis, researchers have identified its involvement in the development of several diseases, including tumors, neurodegenerative diseases, and viral infections (Wang Z. et al., 2022; Paik et al., 2021). Numerous studies have shown that NLRP3 inflammatory mediated pyroptosis plays an important role in IVDD and that inhibition of pyroptosis in the NP can mitigate IVDD progression (Ge et al., 2022; Gong et al., 2023). Futhermore, nutrition is primarily supplied to the IVD through the cartilage endplate, a thin layer of hyaline cartilage between the vertebral body and IVD (Yuan et al., 2019). The EP is the pathway for nutrients and protects the adjacent avascular IVD from degeneration by maintaining its integrity and function. In addition, the EP plays critical functions in maintaining stress distribution. CEP dysfunction, including apoptosis, calcification, and porosity changes in chondrocytes, is thought to be the main manifestation that accelerates IVDD (Yang et al., 2020). Our previous study found that significant structural remodeling of the cartilage endplate occurs in a rat model of osteoporosis combined with adjacent segment disc degeneration, and that inhibition of cartilage endplate remodeling ameliorates its degeneration (Sun et al., 2021a).

DM is a chronic metabolic and endocrine disease caused by partial or complete insulin deficiency or cellular resistance to insulin receptors in target tissues, and the sudden increase in the incidence of DM in recent years has caused tremendous mental stress to patients as well as heavy medical burdens on society and families (Li et al., 2024). Approximately 40% of cases of LBP accompany IVDD and much more evidence indicates that DM contributes significantly to the severity of IVDD (Liu et al., 2018). Numerous studies have confirmed that DM is associated with many musculoskeletal disorders, such as synovitis, joint stiffness, osteoarthritis, rheumatoid arthritis, diabetic myasthenia gravis, diffuse idiopathic skeletal hypertrophy, osteoporosis and IVDD (Choi et al., 2022; Majjad et al., 2018). Diabetes-induced non-enzymatic glycosylation of collagen with advanced glycation end-products (AGEs) formation, which could increase collagen cross-linking and be thought to be the pathogenesis of joint-related diseases (Abate et al., 2010). Interestingly, Krishnamoorthy et al. reported that AGEs were found in NP of IVDs and mediated dysregulation of aggregated protein synthesis and EP sclerosis (Krishnamoorthy et al., 2018).

Osteoporosis, a chronic complication of DM, which is a systemic metabolic disease prevalent in postmenopausal and older populations and characterized by reduced bone mass, increased bone fragility, and increased risk of fracture (Scha et al., 2021). Bao et al. (2023) found that the disruption of Wnt signaling triggered by chronic hyperglycemia is involved in the pathogenesis of diabetic osteoporosis. In addition, osteoporosis is not only an important risk factor for complications of DM but also the main inductor of IVDD. The above showed that there is an inseparable relationship between osteoporosis, DM and IVDD.

Ipriflavone (IP) is an isoflavone derivative, which is similar to estradiol in chemical structure. It is currently used clinically to improve primary osteoporosis, prevent brittle fractures, promote fracture healing and other bone metabolic diseases (Hu et al., 2020). One study confirmed that it plays a role in the metabolism bone tissue, improving its density preventing loss, contributes reducing risk fractures (Miedziaszczyk et al., 2023). In addition, IP as a non‐steroidal glucocorticoid receptor antagonist which not only effectively lowers blood glucose levels but also ameliorates diabetic cognitive dysfunction (Nie et al., 2022). Futhermore, Paesa et al. (2021) reported that IP can effectively IP can inhibit the degeneration of osteoarthritis chondrocytes induced by lipopolysaccharide and delay the progression of osteoarthritis.

However, it is still unknown whether IP can inhibit the pyroptosis of NP, the degradation of ECM and whether it can improve the prognosis of osteoporosis in diabetic IVDD. In the present study, a instability of lumbar spine (ILS) and intraperitoneally injecting streptozotocin (STZ) mice model was used to investigate the effect of IP administration on DM with IVDD, providing a basis for the clinical treatment of IVDD in DM patients.

## Materials and methods

### Animals

All experimental protocols were approved by the Institutional Animal Care and Use Committee. In this study, sixty 8-week-old healthy female C57BL/6J mice were purchased from Yi Wei Wo Technology Co., Ltd. (Shijiazhuang, China). The animals were group-housed six per cage under a 12-hour light-dark cycle with access to food and water at a constant temperature of 21°C.

### Experiment design and operation procedures

Adaptive feeding was conducted for 1 week, followed by a sham operation (A skin incision was made and then sutured) or instability of lumbar spine (ILS, established the IVDD model). Instability of lumbar spine was induced by surgical resection of posterior elements including facet joints, supra- and interspinous ligaments. The surgical procedure replicated the previously validated model described by Oichi and colleagues (Oichi et al., 2018). In the surgery group, bilateral facet joints at the L4–L5 level were exposed but not transected. Thenbilateral inferior articular processes were transected by microscissors using a surgical microscope, followed by transection of supra- and interspinous ligaments. All animals were given prophylactic antibiotics (penicillin-G; 40,000 U) for 3 days after the surgery. One week after ILS surgery, the experimental model of DM was established by intraperitoneally injecting streptozotocin (STZ; 60 mg/kg/day/L, freshly prepared, pH 4.5 in citrate buffer) for 5 consecutive days. Forty-eight hours after the STZ injections, mice with blood glucose levels above 200 mg/dL were considered to have DM (Nan et al., 2022). Ipriflavone was used to treat IVDD with DM. The treatment was performed 24 h after the STZ injection and then once a day for 6 weeks in the following groups (n = 12 each): (1) Sham, (2) ILS, (3) STZ, (4) ILS + STZ, and (5) ILS + STZ + IP(200 mg/kg (Guo et al., 2019). After the treatment, the blood glucose and insulin levels were evaluated to determine the fasting levels; subsequently, the L3–L6 segment of the spine was removed. Half of the samples (n = 6, each group) were used for histological, immunohistochemical (L4–L5, n = 6) real-time polymerase chain reaction (RT-PCR), and Western blot (L3–L4, n = 3) analysis. The other half (n = 6, each group) underwent microcomputed tomography (μCT) scanning.

### μCT testing

The SkyScan 1,176 microcomputed tomography system was used to evaluate the L4–L5 segment as previously described (Xiao et al., 2018). NRecon v1.6 software was used to reconstruct images scanned at 50 kV, 450 μA, and 8 μm. We obtained three-dimensional reconstructed images with CTvox v3.0. The CTAn v1.14 and Data Viewer v.1.5 were used to select the vertebrae and EP regions of interest (ROI).

As in our previous studies (Sun et al., 2021a; Sun et al., 2021b), the transverse images of L5 vertebrae were used to measure the vertebral bodies after excluding the cortical bone. The disk height index (DHI) was determined by this formula: anterior disk height + posterior disk height/anterior vertebral bone height + posterior vertebral bone height. A mid-sagittal measurement of IVD and vertebral bone height was performed to calculate the disc height index (DHI) (Lu et al., 1997). ROI for CEPs was restricted to the visible bone plate covering the vertebrae. An overview of the vertebral structure was obtained by measuring the bone volume fraction (BV/TV), trabecular thickness (Tb.Th), trabecular number (Tb.N) and trabecular separation (Tb.Sp) parameters. There were several parameters determined for the EP, including the number of closed pores (Po.N (cl); representing the number of pores with a closed cavity in the endplate structure), open porosity (Po(op); open pore volume over total pore volume), and total pore space volume (Po.V (tot)).

### Histology and immunohistochemistry examinations

A 2-month decalcification in 10% EDTA-2Na was performed after fixation in 10% neutral paraformaldehyde for 48 h. We dehydrated and embedded the paraffin-embedded decalcified samples. The sections were then cut into 6-μm slices for Van Gieson (VG), Alcian blue (Ab), and immunohistochemistry staining. VG and Ab staining were performed using BA408A VG and DG0041Ab kits and The degenerative changes in the L4–L5 segment were assessed using the disc degeneration assessment scoring system described by Masuda et al. (Masuda et al., 2005). This analysis was performed by two independent researchers in a blinded manner.

The expression of aggrecan (1:100; Abcam Inc., United States), A disintegrin and metalloproteinase with thrombospondin motifs 4 (ADAMT4) (1:300; Gene Tex Inc.,United States), matrix metalloproteinase-13 (MMP-13) (1:500; Boster Co.,Ltd., Wuhan, China), Runt-related transcription factor 2 (Runx2) (Abcam, ab76956, 1:300), and Osterix (Abcam, ab22552,1:300) in the NP or EP was assessed by IHC. Each section was deparaffinized, rehydrated, and immunostained in succession, followed by 30 min of incubation by pancreatin to retrieve the antigens. Afterward, 3% H_2_O_2_ was used to block endogenous peroxidase activity, and the samples were incubated in primary antibodies overnight at 4°C. The next day, the samples were washed with Tris-buffered saline for 15 min and incubated in biotin-labeled goat anti-rabbit IgG (ZSGB-BIO, PV-6000). The chromogenic substrate, diaminobenzidine (ZSGB-BIO Corp, China) was used for color development, and hematoxylin was used for counterstaining.

### RT-PCR analysis

A Gene Amp 7,700 Sequence Detection System (Applied Biosystems, Foster City, CA, United States) and SYBER^®^ Premix Ex Taq™II kit (Takara, Kusatsu, Japan) were used for the RT-PCR. The primers for the selected genes are listed in Table 1. GAPDH was used as an endogenous control. The changes in relative mRNA transcript levels were calculated using the 2 (-Delta C(T)) method as previously described (Livak and Schmittgen, 2001).

**TABLE 1:** The effect of IP on blood glucose, blood insulin levels and HbA1c among all groups.

Group	Blood glucose (mmol/L)	Blood insulin levels (μIU/mL)	HbA1c (%)
Sham	5.17 ± 0.18	40.61 ± 1.36	4.58 ± 0.09
ILS	6.19 ± 0.40^**^	39.67 ± 2.20	5.10 ± 0.20^**^
STZ	19.83 ± 0.73^**^	25.97 ± 0.82^**^	11.91 ± 0.37^**^
ILS + STZ	24.38 ± 0.75^**^	23.86 ± 1.49^**^	14.19 ± 0.38^**^
ILS + STZ + IP	8.14 ± 0.19^##^	34.46 ± 0.97^##^	6.07 ± 0.10^##^

Compared with the Sham group, **P < 0.001. Compared with the ILS + STZ, group, ##P < 0.001. Data are expressed as mean ± SD (n = 12).

### Western blot analysis

The NP of IVDs were collected and dissolved in a radioimmunoprecipitation analysis buffer using a cocktail of protease inhibitors. The protein lysate was electrophoresed with sodium dodecyl sulfate-polyacrylamide gel and transferred to nitrocellulose membranes. Next, 5% bovine serum albumin was used to block the membranes at 4°C for 2 h. The membranes were subsequently incubated with the following primary antibodies at 4°C overnight: nod-like receptor protein-3 (NLRP3) (1:1,000, Abcam), caspase1 (1:500, Abcam), apoptosis-associated speck-like protein (ASC) (1:500, Abcam), and N-gasdermin D (N-GSDMD) (1:1,000, Abcam). After washing with TBST, the membranes were incubated with HRP-conjugated secondary antibodies at a dilution of 1:2000 at 4°C for 4 h. The proteins were visualized using an enhanced chemiluminescence kit (Merck Millipore, Billerica, MA, United States) and an imaging system (Bio Spectrum 600; UVP, Upland, CA, United States).

### Statistical analysis

All data were analyzed using SPSS software (SPSS, Chicago, IL, United States), and the results were expressed as the mean ± standard deviation (SD). The Shapiro-Wilk test for normality and Bartlett’s test for homogeneity of variance were performed. One-way analysis of variance (ANOVA) and Fisher’s protected least significant difference test were used to determine the statistically significant differences between the groups. The results of the radiography scores were analyzed using the KruskalWallis test. P < 0.05 was considered to indicate statistical significance.

## Results

### IP inhibits the rise of blood glucose level and improves DM

**FIGURE 1:**
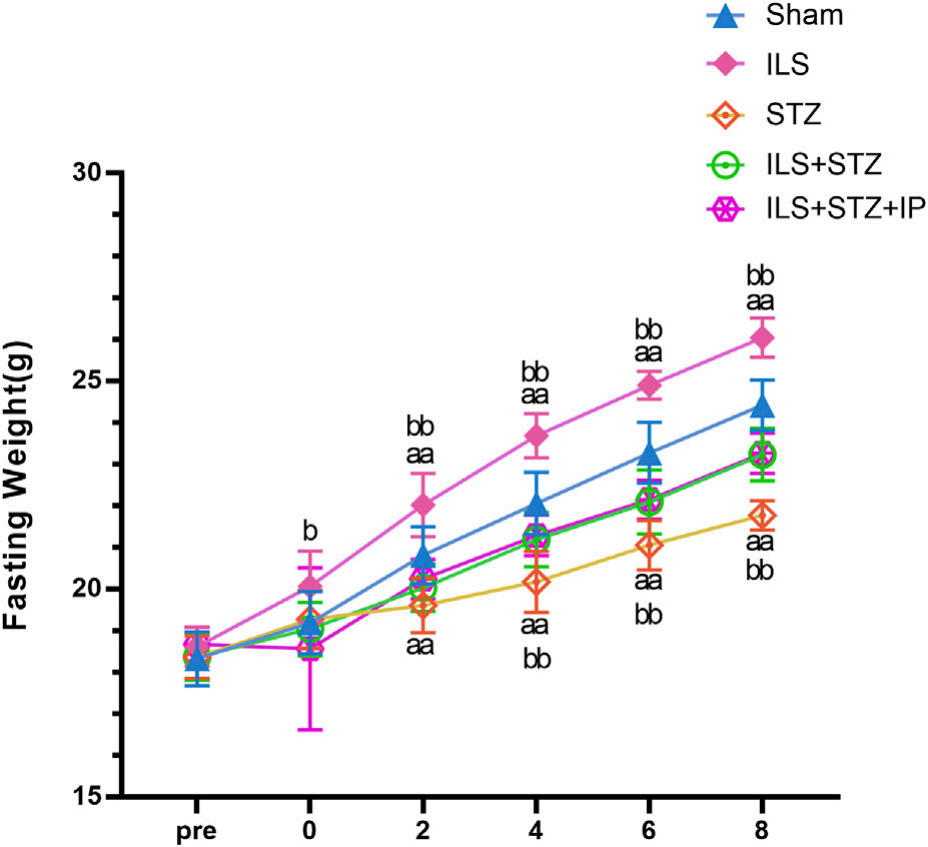
Changes in fasting weight among all groups. Fasting weight in the Sham, ILS, STZ, ILS + STZ and ILS + STZ + IP groups. Before operation (Pre); after operation and streptozotocin injection (0), IP was administered from the first week to the eighth week on alternate days. Note: Compared with the Sham group, ^a^ *P* < 0.05 and ^aa^ *P* < 0.01. Compared to ILS + STZ group, ^b^ *P* < 0.05 and ^bb^ *P* < 0.01.

The body weight of the mice was measured every 2 weeks. As shown in Figure 1, the weight of the mice in all groups increased over time. However, from week 2 to week 8, the weight increase was most significant in ILS group. Compared with the Sham group, the blood glucose and HbA1c levels of mice in the ILS, STZ, and ILS + STZ groups were higher but the blood insulin level were lower in the ILS, STZ, and ILS + STZ groups, especially in the ILS + STZ group. This trend was significantly reversed after the IP treatment (Tables 1, 2). These results confirmed that IP effectively controlled blood glucose levels and had anti-diabetic effects.

**TABLE 2:** Sequences of primers used for RT-PCR

Gene	Forward primer (5′-3′)	Reverse primers (5′-3′)
GAPDH	GGG​GAG​CCA​AAA​GGG​TCA​TCA​TCT	GAG​GGG​CCA​TCC​ACA​GTC​TTC​T
NLRP3	TTG​AAG​AGG​AGT​GGA​TAG​GT	GGTGTAGCGTCTGTTGAG
ASC	AACTTGACAGCGGATGAG	CTC​CAG​ACT​CTC​CAT​AAT​CT
Caspase1	TGG​ATT​GCT​GGA​TGA​ACT​T	CTG​ATG​GAC​CTG​ACT​GAA​G

### IP inhibited the endplate osteochondral remodeling and vertebral osteoporosis

**FIGURE 2:**
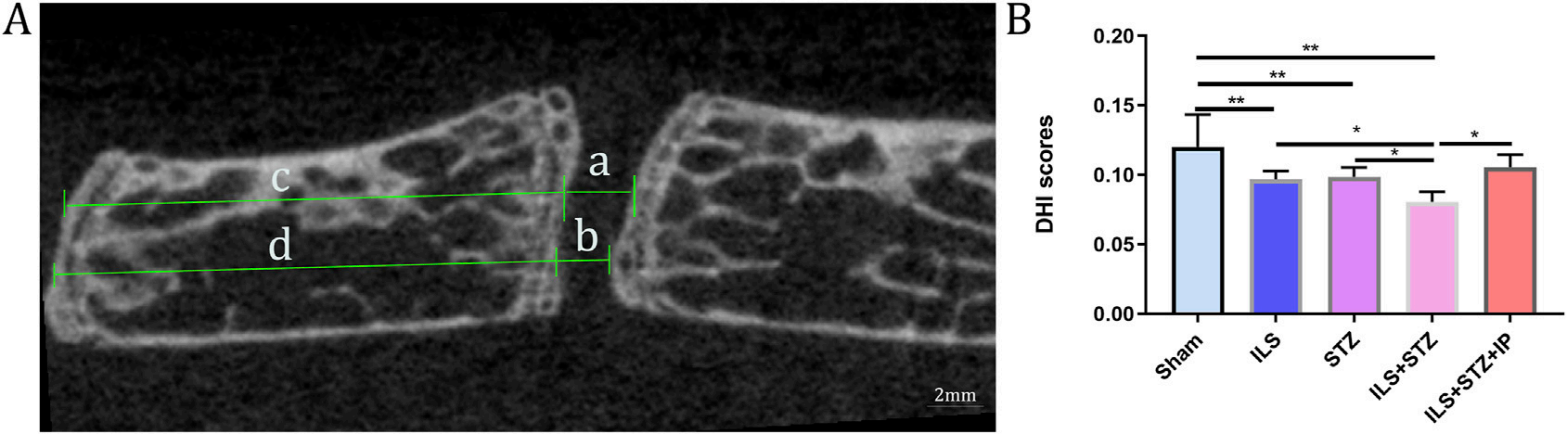
Representative micro-CT images were used to quantify the DHI of L4-L5 of the IVD, calculated based on measurements of the adjacent L4 vertebra. **(A)** The following equation was used to calculate the DHI: DHI= (a+b)/(c + d). **(B)** DHI scores results. Note: *P < 0.05, **P < 0.01; scale bars = 2 mm as indicated.

**FIGURE 3:**
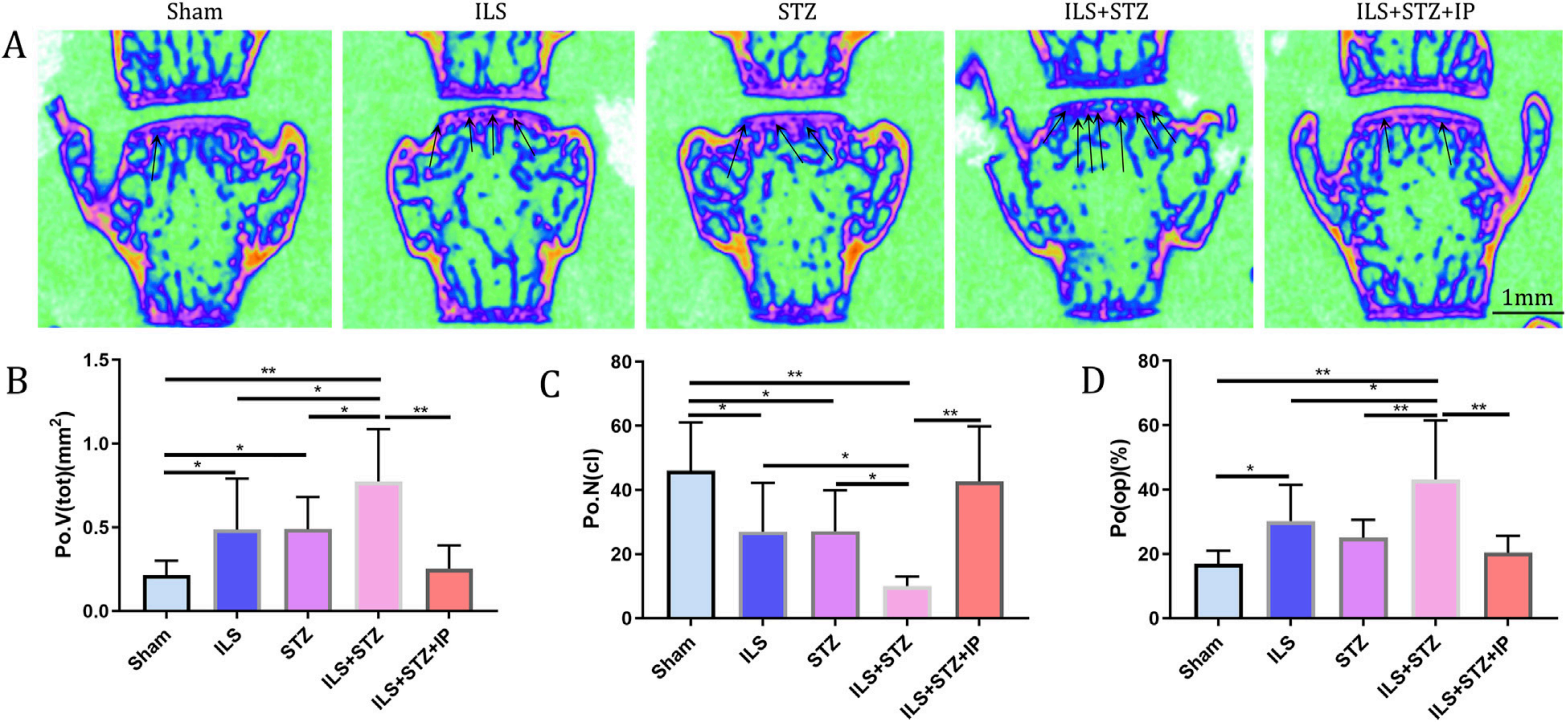
Changes in the microarchitecture of the L4-L5 caudal EP. Representative coronal images and parameters of the caudal EP in each group. **(A)** The increase in cavities in the ILS and ILS + STZ groups (black arrow) indicates osteochondral EP remodeling. **(B–D)** Compared with the Sham group, a markedly lower number of closed pores (Po.N (cl)), higher open porosity (Po(op)), and total pore volume (Po.V (tot)) occurred in the ILS and ILS + STZ groups. Compared with the ILS + STZ group, there was a significantly higher number of Po.N (cl), and decreased Po(op) and Po.V (tot) in the ILS + STZ + IP group. Note: *P < 0.05, **P < 0.01; scale bars = 1 mm as indicated.

We first examined the space between lumbar vertebral bodies since a narrowed IVD space is characteristic of IVDD. As shown in Figure 2, the midsagittal scanning images revealed that the ovariectomy groups demonstrated a narrower IVD between L4 and L5 than the Sham group, especially in the ILS + STZ group, However, greater than that of the ILS + STZ + IP group the DHI was significantly increased compared with ILS + STZ group. Then we examined the parameters of L4-L5 caudal EP, which demonstrated numerous cavities in the ILS and ILS + STZ groups. As shown in Figure 3, the trabeculae were sparser and the canal between trabeculae was wider in the ILS and ILS + STZ groups versus the Sham group. In addition, a large number of trabeculae were absent in the ILS + STZ group. However, these effects were reversed in the ILS + STZ + IP group. μCT evaluation of the L4-L5 caudal EP showed that compared with the Sham group, the ILS and ILS + STZ groups had a significantly lower Po.N along with a higher Tb. Sp. Compared with the ILS + STZ group, a significantly higher Po.N but lower Tb. Sp were seen in the ILS + STZ + IP group.

**FIGURE 4:**
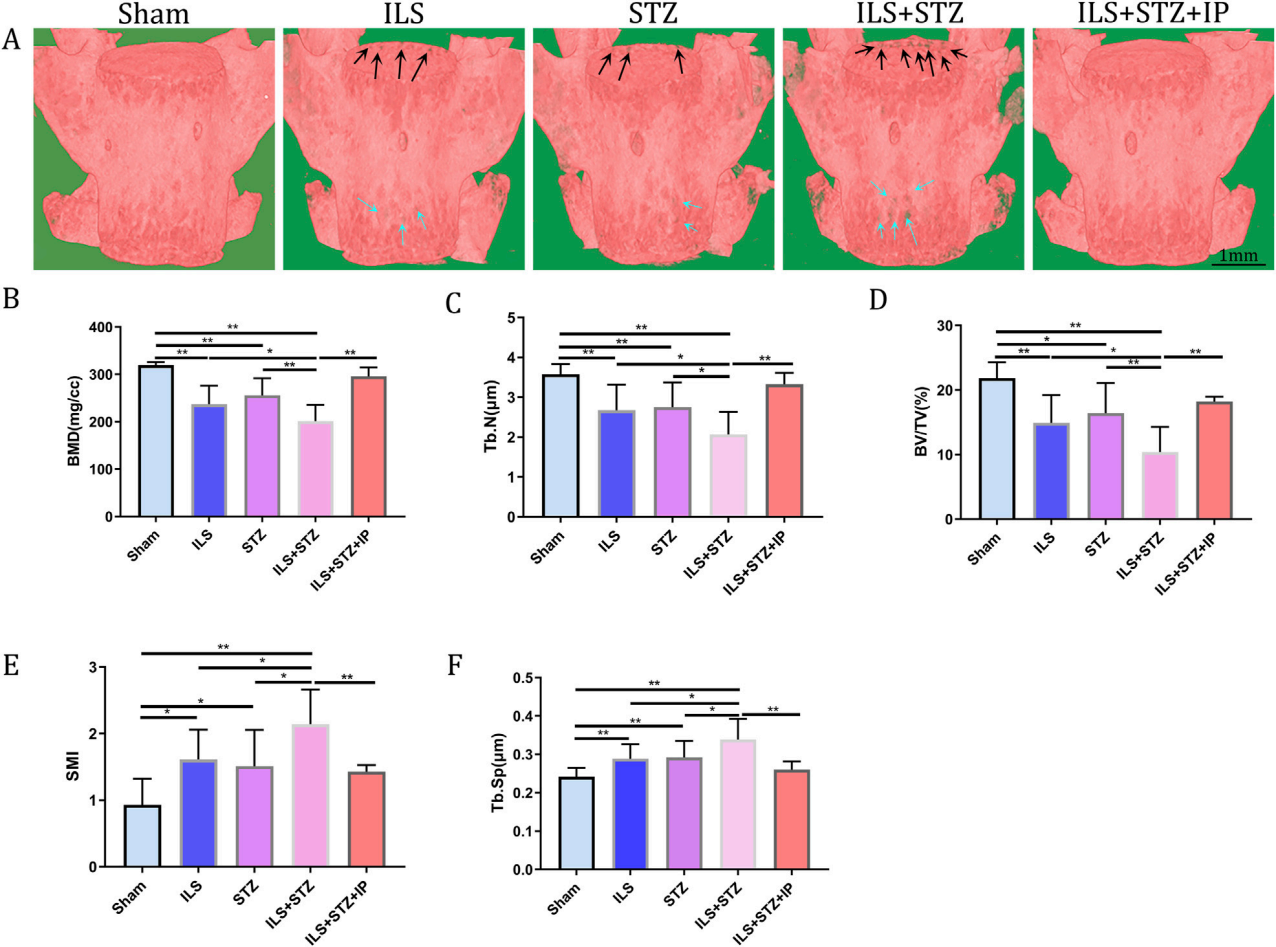
Representative μCT images of L5. **(A)** Compared with the Sham group, the trabeculae were sparser and the width of the canal between trabeculae was greater in the ILS and ILS + STZ groups; a large area of bone trabeculae was missing in the ILS + STZ group. These trends were effectively suppressed in the ILS + STZ + IP group. The parameters of L5 trabecular bone. **(B–F)** The results of L5 vertebral BMD, bone volume (BV)/total volume (TV), trabecular number (Tb.N; mm^-1^) and trabecular separation (Tb.Sp; μm) measurements in different groups. Note: *P < 0.05, **P < 0.01; scale bars = 1 mm as indicated.

In addition, the ROI of the vertebrae was marked (Figure 4). The results of μCT analysis confirmed that the ILS and ILS + STZ groups had a significantly higher Tb. Sp and a lower Tb.N, BMD, and BV/TV than the Sham group. Importantly, these results provided valuable insight into the role of osteoporosis in cartilaginous EP resistance and vertebral degeneration in IVDD. The above pathological changes were effectively improved after IP treatment.

### IP inhibited the sclerotic and osteogenic transdifferentiation of the cartilage EPs

**FIGURE 5:**
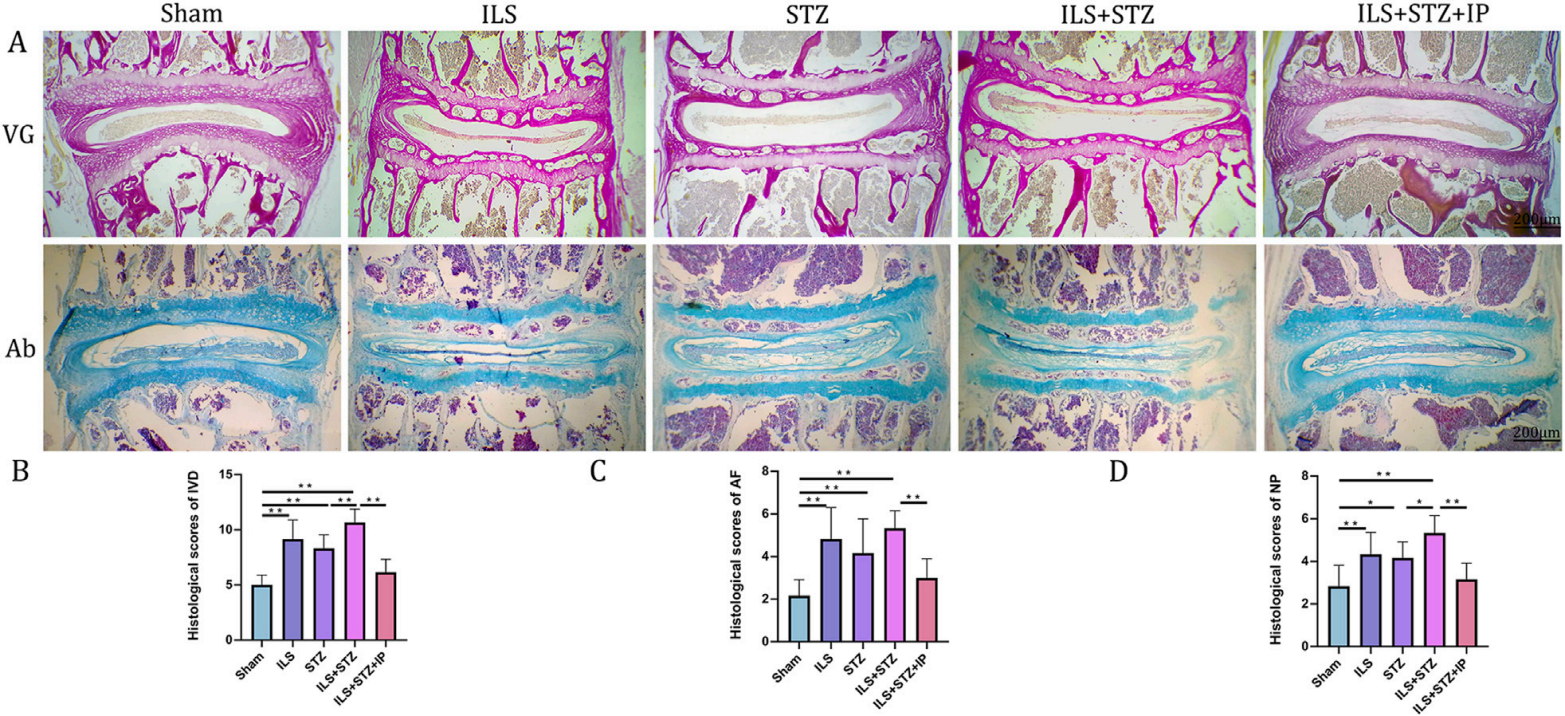
Histological illustration of the L4-L5 IVD in the different groups. **(A)** VG and Ab staining of L4-L5. **(B)** Histological scores of the L4-L5 of the IVD in the different groups. **(C)** Histological scores of the L4-L5 of the AF in the different groups. **(D)** Histological scores of the L4-L5 of the NP in the different groups. Note: *P < 0.05, **P < 0.01; scale bars = 200 μm.

**FIGURE 6:**
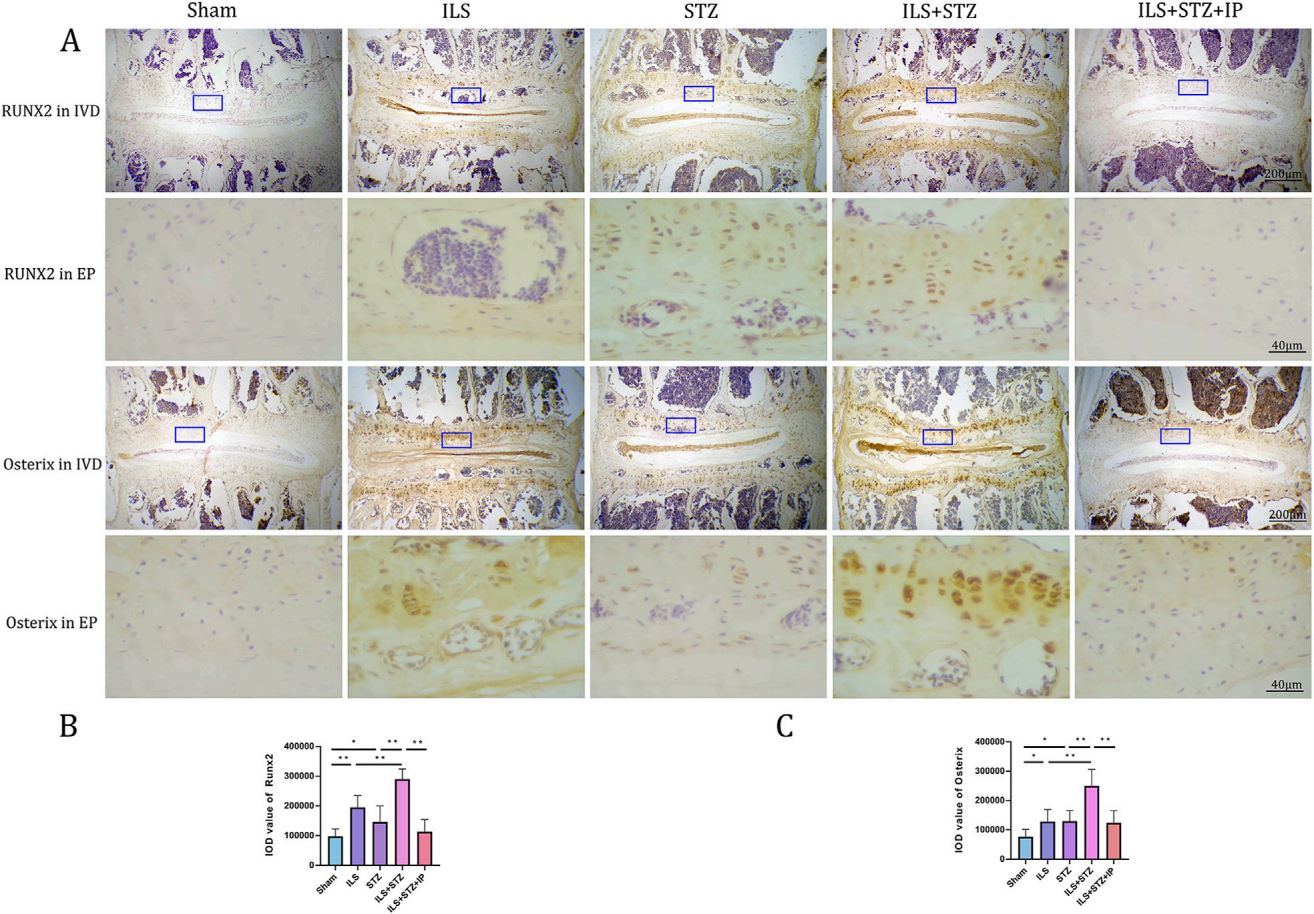
Immunohistochemistry assay for Osterix and Runx2 in the IVD among all groups **(A)**. **(B,C)** Immunohistological analysis of NP showed that the protein expression levels of Osterix and Runx2 in the ILS + STZ + IP group were lower than those in the ILS + STZ group. Note: *P < 0.05, **P < 0.01; scale bars = 200 μm as indicated.

Based on the radiological results, we next explored the effect of cartilaginous EP sclerosis in IVDD. As shown in Figure 5, compared with the Sham group, an obvious increase in EP calcification was observed in the ILS and ILS + STZ groups. In agreement with the μ-CT results, the formation of bone marrow cavities also increased in the EP region. Furthermore, two osteogenic transcription factors, Runx2 and Osterix, were detected in EP chondrocytes (Figure 6). In the ILS and ILS + STZ groups, the protein expression levels of Runx2 and Osterix were substantially increased in comparison with those in the Sham group, especially in the ILS + STZ group. The above changes were reversed after treatment with IP. These findings suggest that osteoporosis accelerates IVDD through cartilage EP sclerosis and osteogenic transdifferentiation in diabetic mice and that IP canreverse this trend.

### IP inhibited the pyroptosis of NP cells in diabetic IVDD

**FIGURE 7:**
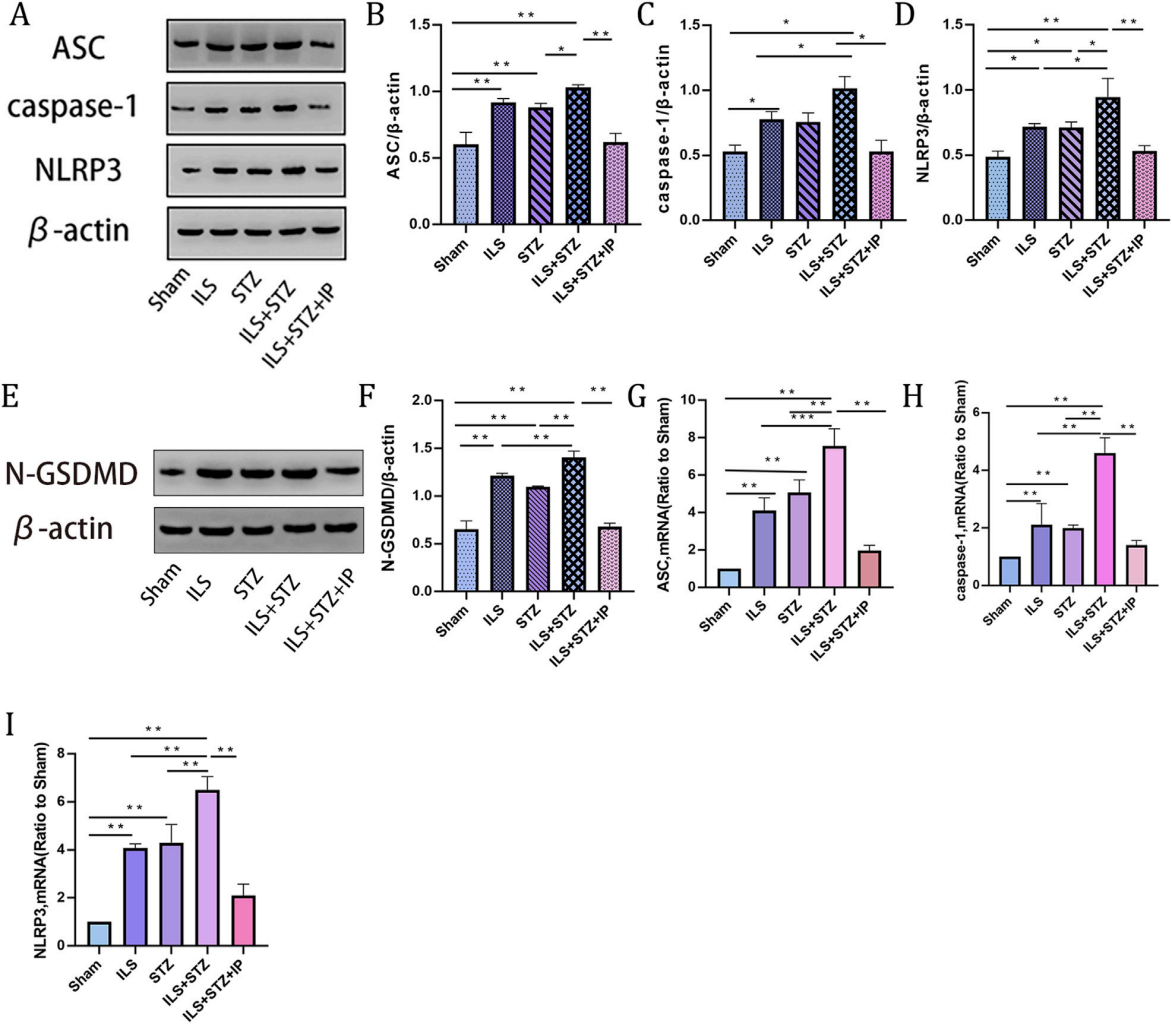
Western blot and RT-PCR of pyroptosis markers in NP across all groups. **(A)** Representative Western blotting result showing ASC, caspase 1 and NLRP3 expression levels. **(B–D)** Quantitative analysis of ASC, caspase 1 and NLRP3 protein expression levels. **(E)** Representative Western blotting result showing N-GSDMD expression levels. **(F)** Quantitative analysis of N-GSDMD protein expression. **(G–I)** Quantitative analysis of ASC, NLRP3, and N-GSDMD mRNA expression. Note: *P < 0.05, **P < 0.01.

The protein and mRNA expression levels of pyroptosis markers were detected to observe the pyroptosis of NP cells in each group. As shown in Figure 7, compared with the Sham group, the protein levels of NLRP3, ASC, N-GSDMD and caspase1 increased significantly, especially in the ILS + STZ group. In addition, the mRNA expression levels of NLRP3, ASC, and Caspase1 were compatible with the Western blot results. However, the above expression trend was significantly reversed in the ILS + STZ + IP group. The above results confirm that IP can effectively inhibit NP cell pyroptosis and delay the occurrence and development of diabetic IVDD.

### IP disrupted the degradation of the cartilage extracellular matrix

**FIGURE 8:**
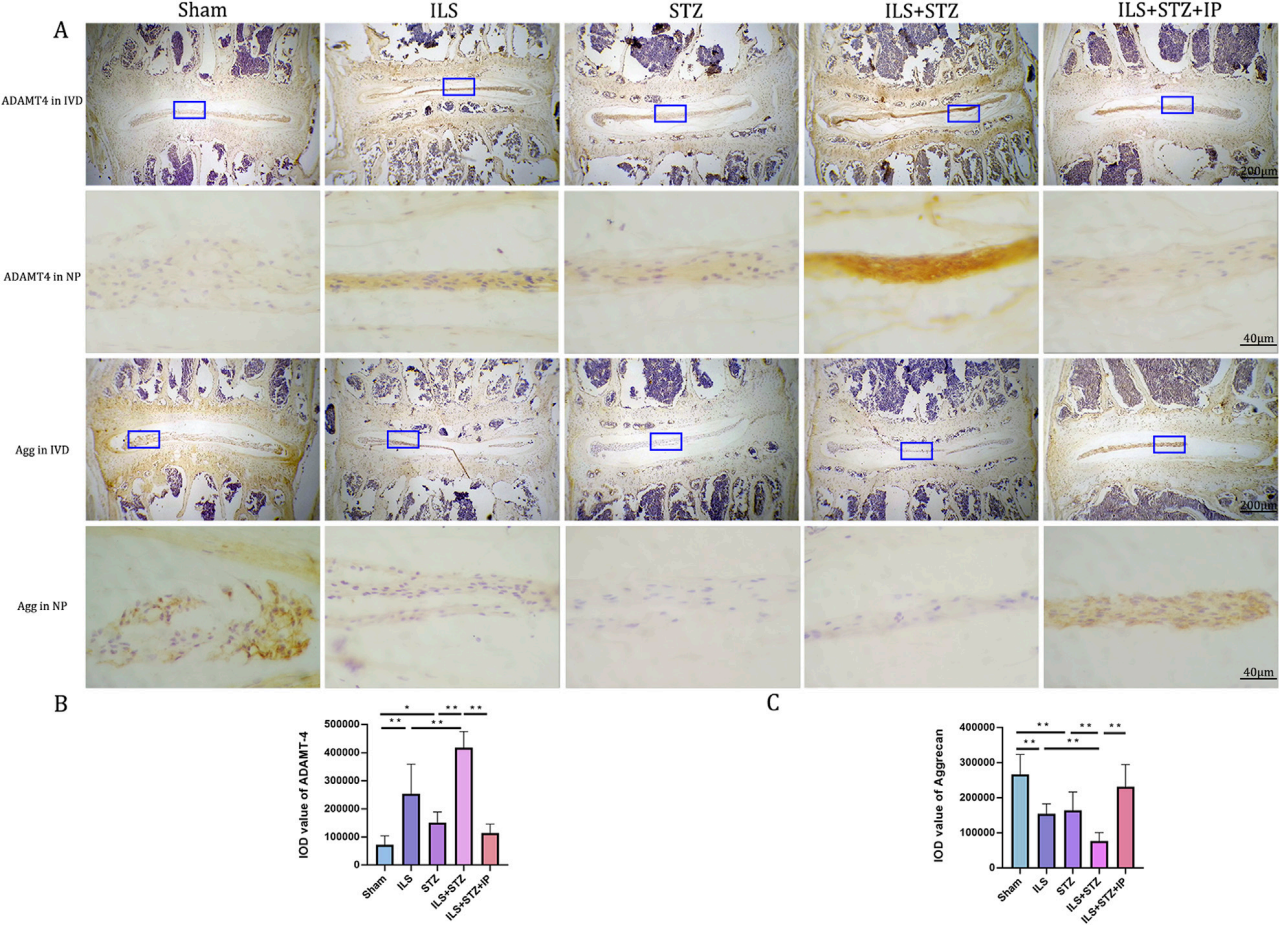
**(A)** Representative immunohistochemistry images of ADAMTS-4 and Aggrecan staining across all groups. **(B, C)** Quantitative analysis of ADAMTS-4 and Aggrecan protein expression levels in the NP. Immunohistological analysis showing that the protein expression level of Aggrecan in the ILS+STZ+IP group was higher and this of ADAMTS-4 were lower than this in the ILS+STZ group. Note: *P < 0.05, **P < 0.01; scale bars = 200 μm and 40 μm as indicated.

**FIGURE 9:**
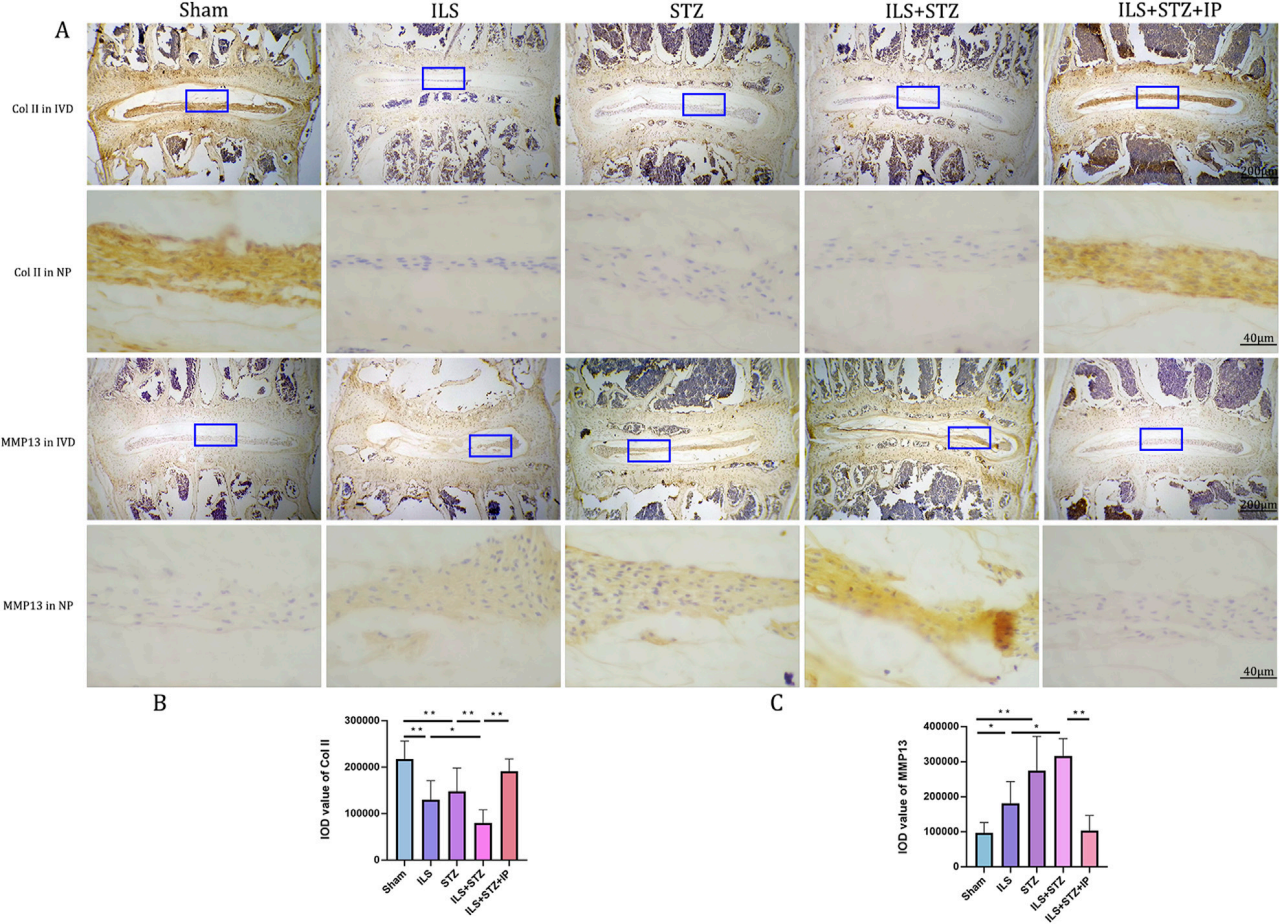
**(A)** Representative immunohistochemistry images of ColII and MMP-13 staining across all groups. **(B, C)** Quantitative analysis of ColII and MMP-13 protein expression levels in the NP. Immunohistological analysis showing that the protein expression level of ColII in the ILS+STZ+ IP group was higher and this of MMP-13 was lower than those in the ILS+STZ group. Note: *P < 0.05, **P < 0.01; scale bars = 200 μm as indicated.

Degradation of the extracellular matrix is considered a major mechanism underlying IVDD. We performed VG, Ab, and immunohistochemical staining to detect differences in the tissue structure of IVDs and changes in cartilage extracellular matrix among the groups (Figure 5). The IVD structure was intact in the Sham group. Specifically, NP was accompanied by rich gel-like tissue (extracellular matrix), and AF was arranged regularly and tightly. The EP contained chondrocytes. However, IVDD occurred in the ILS, STZ, and ILS + STZ groups to varying degrees. In addition, Ab staining revealed that cartilage EP, AF, NP, and the extracellular matrix around the NP (blue) were more prominent in the Sham group and, significantly reduced in the ILS, STZ, and ILS + STZ groups. The above trends were effectively improved after IP treatment. IVD scores confirmed these histological findings. As shown in Figures 8, 9, in the Sham group, EP with weak immunoreactivity for MMP13 and ADAMTS-4 and strong immunoreactivity for Agg and Col II was observed. By contrast, the ILS and ILS + STZ groups showed strong MMP13 and ADAMTS-4 immunoreactivity and weak Agg and Col II immunostaining. This effect was more pronounced in the ILS + STZ group. However, this trend was reversed by IP treatment. These results provide important insight into the role of IP in treating to cartilaginous EP matrix degeneration in IVDD.

## Discussion

Globally, Low back pain is largely caused by IVDD, which is also responsible for spinal disorders such as disc herniation and lumbar spinal stenosis (Ansuategui Echeita et al., 2020). With the rapid development of the world’s economy, people’s standard of living has increased dramatically. Their prevalence of diabetes is increasing worldwide. Many people develop both DM and IVDD (Li et al., 2022). It has been shown that DM is an important causal factor in disc degeneration, and apoptosis and senescence are increased in diabetic nucleus pulposus (NP) tissues (Yu et al., 2023). In this study, we used a DM and IVDD mouse model that simulates the IVDD pathology of diabetic patients and demonstrated that the dual effects of ILS and DM aggravated the degradation of cartilage extracellular matrix, NP pyroptosis and the bone microstructure of vertebra and EP in mice. As expected, IP significantly inhibited the above pathologic changes and delayed the development of IVDD.

Different from the anti-osteoporosis drugs, IP is an isoflavone derivative that is chemically similar to estradiol, can significantly improve osteoporosis and play an important protective role in a variety of bone metabolic diseases, while having the advantage of having few side effects (Gao et al., 2018). In addition, IP as a non‐steroidal glucocorticoid receptor antagonist that can not only control blood glucose level but also ameliorates diabetic cognitive impairment in mice (Nie et al., 2022). Some studies have shown that IPhas anti-inflammatory properties, promoting blood circulation and anti-oxidative stress in the treatment of neurological diseases (Yassa et al., 2020; Calis et al., 2020). In the present study, it was found that IP not only significantly reduced blood glucose levels and inhibited the pyroptosis of NP but also retarded the degradation of cartilage extracellular matrix and improved the osteoporosis of EP and vertebrae, which could effectively delay the progression of IVDD.

Anatomically, as an important component structure of the IVD, the EP is a layered composite of semi-porous thickened cancellous bone and hyaline cartilage that transmits compressive loads to the spine and transfers water, nutrients and waste products into and out of the IVD (Wang Y. et al., 2022). Consistent with previous research, we found that the dual effects of estrogen deficiency and diabetes may induce more severe microstructural changes in the EP region, much more bone marrow and sclerosis formed. But, the above pathological change was effectively improved after IP treatment. Furthermore, diabetic ILS mice demonstrated reduced disc height, altered vertebrae microstructure and increased extracellular matrix degradation (Chen et al., 2018). Our data revealed that there was a negative correlation between EP porosity, EP sclerosis, extracellular matrix degeneration, the degree of vertebrae osteoporosis and DHI in the IVDD with DM mouse model. Which suggested that IVDD is caused by a degenerative cycle of biomechanics, microarchitecture, and extracellular matrix. Notably, the μCT analysis in the present study revealed that DM further exacerbate the degeneration of the bone microarchitecture, which accelerated IVDD. We also observed osteoporosis in the diabetic mice, which confirms that DM is an important causative factor in the development of osteoporotic IVDD. And the above pathological changes were effectively improved after IP treatment.

In previous studies, degeneration of the NP was suggested to be the main component of IVDD. Hence, the relatively large number of studies on the NP and the minimal focus on the AF and EP (Shao et al., 2021). The EP is the main access point for nutrients and blood and acts as a cushion for mechanical loads; thus, degeneration of the EP is of increasing interest to scholars. Degeneration of the EP, mainly sclerosis, is the earliest pathological change in IVDD and its early detection and active control is of great clinical importance in the treatment of IVDD. In this study, results from VG and Ab staining exhibited that EP sclerosis was accompanied by the degradation of the EP cartilage matrix and alteration of microstructure of EP. In addition, the protein levels of Runx2 and its downstream effector Osterix in the EP were significantly elevated in the ILS, STZ, and ILS + STZ groups compared with those in the Sham group. Runx2 and Osterix are considered vital regulators of the transdifferentiation of chondrocytes to osteogenic precursor cells (Dalle et al., 2022). Thus, we infer that EP sclerosis accelerated expression of osteogenic related transcription factors in diabetic mice during IVDD. However, these trends were effectively suppressed by the administration of IP.

Furthermore, unlike other types of cell death, pyroptosis is a newly identified inflammatory programmed cell death mechanism mediated by inflammatory vesicles, whose distinctive features include pore formation on the plasma membrane, cell swelling and rupture, and subsequent release of inflammatory cytokines and intracellular substances (Humphries et al., 2020). Meanwhile, some studies have confirmed a significant increase in pyroptosis-related marker proteins found in degenerating IVD tissues in humans and mice, further confirming that pyroptosis may play a large role in the pathological process of IVDD (Fu et al., 2021; Zhang J. et al., 2020). Consistent with other scholarly studies, our latest findings also demonstrated a significant increase in the expression of pyroptosis-related proteins, such as NLRP3, caspase-1, and GSDMD in the ILS, STZ, and ILS + STZ groups, especially in the ILS + STZ group. However, IP reversed this effect, and the conditions improved significantly.

In the normal IVD, the NP is a highly hydrated, gelatinous structure primarily comprising notochord cells and extracellular matrix (ECM) components produced by NP cells, including col II and proteoglycans, which are considered key factors in resisting axial stresses and strains in the spine, and degeneration of NP tissue is also key contributor to the IVDD (Zhao et al., 2024). Considering that degradation of the extracellular matrix is an important aspect of IVDD pathogenesis, we correlated the ECM of the NP in the present study. Ab and immunohistochemical staining analyses were performed. Compared with the Sham group, in the ILS, STZ, and ILS + STZ groups, the expression levels of MMP13 and ADAMT-4 were significantly increased, while the expression levels of Col2 and Agg were significantly decreased, especially in the ILS + STZ group. However, it is gratifying to note that ECM degradation is effectively ameliorated by IP treatment in DM with IVDD mice. In this context, one limitation of the present study is lack of positive control and *in vitro* study. Future studies should focus on explore the relevant molecular mechanisms in the IVDD and DM. Under the current era where the incidence of DM combined with IVDD is increasing annually, this study innovatively employs a DM-combined IVDD mouse model to predict human IVDD characteristics, including aging and changes in estrogen levels. Meanwhile, IP was utilized to intervene in disease progression, achieving satisfactory therapeutic outcomes in this study. However, as this research is based on animal experiments, there may be certain limitations in its clinical applicability, necessitating further validation through subsequent clinical studies. In future research, we will further explore the correlation between this model and human IVDD to enhance the applicability of the findings.

**FIGURE 10:**
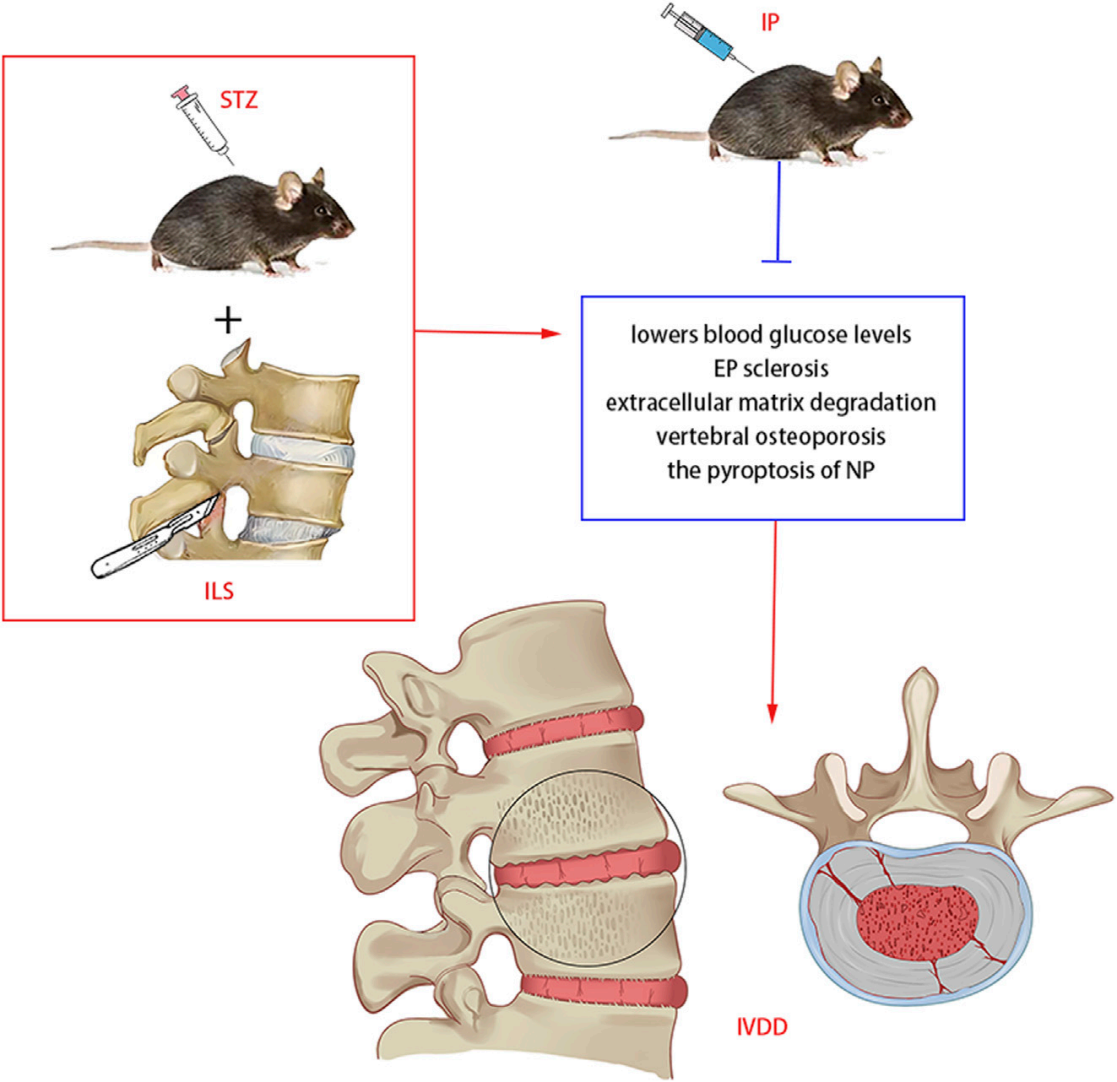
Potential mechanism of IP in diabetic IVDD treatment in mice. IP lowers blood glucose and blood insulin levels, reduces EP sclerosis, improves the degradation ofthe ECM, suppress vertebral osteoporosis and inhibits the pyroptosis of NP.

Overall, we demonstrated for the first time that DM with IVDD induced by ILS and STZ in a mice model could be retarded by intraperitoneally IP injections (Figure10). And we found that IP can effectively lowers blood glucose and blood insulin levels. In addition, the protective effects of IP primarily acted by reducing EP sclerosis, improving the degradation of the ECM, maintaining the vertebral trabecular microarchitecture and inhibiting the pyroptosis of NP. These findings could provide a basis for a novel therapeutic strategy for ASDD and DM.

## Data Availability

The original contributions presented in the study are included in the article/Supplementary Material, further inquiries can be directed to the corresponding authors.

## Glossary

IP: Ipriflavone
IVDD: Intervertebral disc degeneration
DM: Diabetes mellitus
ILS: Instability of lumbar spine
STZ: streptozotocin
BMD: Bone mineral density
AGEs: advanced glycation end-products
Ab: Alcian blue
Micro-CT: Microcomputed tomography
DHI: Disk height index
Agg: Aggrecan
MMP-13: Metalloproteinase-13
ADAMTS-4: A disintegrin and metalloproteinase with thrombospondin motifs 4
bcl-2: B cell lymphoma-2
bax: BCL2-associated X
ROI: Region of interest
BV/TV: Bone volume fraction
Tb.N: Trabecular number
Tb.Sp: Trabecular separation
AF: Annulus fibrosus
EP: Endplates
NP: Nucleus pulposus
VG: Van Gieson
ECM: Extracellular matrix
Runx2: Runt-related transcription factor 2
Po.N(cl): the number of closed pores
Po(op): open porosity
Po.V(tot): total pore space volume
NLRP3: nod-like receptor protein-3
ASC: apoptosis-associated speck-like protein
N-GSDMD: N-gasdermin D
